# Integrated bioinformatic analysis identified a novel prognostic pan-programmed cell death signature for bladder cancer

**DOI:** 10.3389/fimmu.2022.1030097

**Published:** 2022-11-23

**Authors:** Lusi Zhang, Mou Peng

**Affiliations:** ^1^ Department of Urology, Second Xiangya Hospital of Central South University, Changsha, Hunan, China; ^2^ Department of Ophthalmology, Second Xiangya Hospital of Central South University, Changsha, Hunan, China; ^3^ Key Laboratory of Diabetes Immunology (Central South University), Ministry of Education, National Clinical Research Center for Metabolic Disease, Changsha, China

**Keywords:** pan-PCD, bladder cancer, immunotherapy, cell death, chemotherapy

## Abstract

Programmed cell death (PCD) refers to a molecularly regulated form of cell death that functions as an essential anticancer defense mechanism and serves as a target of anticancer therapies. Multiple types of PCD comprehensively regulate tumorigenesis and tumor progression and metastasis. However, a systemic exploration of the multiple types of PCD in cancers, especially bladder cancer, is lacking. In this study, we evaluated the expression pattern of genes associated with multiple types of PCD in bladder cancer using the “ssGSEA” method and conceptualized the multiple types of PCD as being collectively involved in “Pan-PCD”. Based on the differentially expressed genes related to Pan-PCD, we developed a Pan-PCD-related prognostic signature (PPRPS) to predict patient prognosis *via* univariate and multivariate Cox regression analysis. The PPRPS is an independent prognostic factor, and the AUC (Area Under Curve) for 3-year overall survival was 0.748. Combined with age and stage, PPRPS displayed excellent predictive ability. Based on the PPRPS, higher levels of immune cell infiltration, tumor microenvironment, and immune checkpoint molecules were observed in the high-PPRPS group. Furthermore, PPRPS enabled accurate risk prediction for metastatic urothelial carcinoma after anti-PD-L1 monoclonal antibody treatment. Patients in the high-PPRPS group had poor prognoses. Docetaxel, staurosporine, and luminespib were identified as potentially effective drugs for high-PPRPS bladder cancer patients. In summary, we developed the Pan-PCD signature to improve the accuracy of bladder cancer prognostic predictions and to provide a novel classification method to guide treatment selection.

## Introduction

Bladder cancer is one of the most common cancers and represents the 13th most deadly malignancy worldwide (1). Among them, urothelial cell bladder cancer accounts for 90-95% of all bladder cancer cases and is the most prevalent male genitourinary cancer with substantial morbidity (1). The incidence of bladder cancer is steadily increasing, especially in developed countries (2). In 2018, an estimated 550,000 people were diagnosed with bladder cancer worldwide according to GLOBOCAN data, accounting for approximately 3% of all newly diagnosed cancers (3). As the most common type of urological system tumor, bladder cancer is associated with a poor treatment effect due to its high recurrence (4) despite the availability of multiple therapies.

Programmed cell death (PCD) is a dedicated form of molecularly regulated cell death that includes apoptosis, necroptosis, ferroptosis, pyroptosis, autophagy, cuproptosis, and other types of programmed cell death, which is in contrast with accidental cell death (5). And PCD in mammalian cells can usually be delayed or accelerated by pharmacological or genetic interventions (6). PCD has become a critical and popular topic in the cancer research field in recent years. On the one hand, escape from programmed cell death is integral to tumorigenesis, tumor immune evasion (7), and metastasis (8). Various cancer therapies promote tumor cell death (9). On the other hand, inhibiting immune cell death enhances the effects of immunotherapy (10). Furthermore, diverse PCD-related microenvironments may direct lineage commitment in certain types of cancers (11). Physical, chemical, and mechanical insults often lead to accidental cell death (6), which manifests as necrosis and terminates with cell corpse disposal in the absence of obvious phagocytic and lysosomal involvement (12). Accordingly, the PCD environment may act as a potential therapeutic target that recruits immune cells, regulates cell migration, and establishes tumorigenesis and metastasis. The multiple types of PCD can be involved in the definition “Pan-PCD”. The identification of a pan-PCD signature as well as its prognostic effect and treatment characteristics within specific types of cancers is a subject of interest that has not been extensively addressed.

Recently, an increasing number of studies have demonstrated that PCD is tightly correlated with tumorigenesis and treatment (13), especially in bladder cancer. A phase I/II clinical trial on the safety and efficacy of alpha1-oleate treatment in nonmuscle invasive bladder cancer has been reported, and treated tumors exhibited apoptosis and inhibition of the expression of cancer-related genes (14). For example, the Bcl-2 inhibitor ABT-737 can induce MLKL-mediated necroptosis by upregulating RIPK3 expression in bladder cancer (15). OTUB1 expression is markedly elevated in human bladder cancers; knockdown of OTUB1 expression diminishes endogenous SLC7A11, which subsequently enhances ROS-mediated ferroptosis in bladder cancer cell lines (16). Pyroptosis has also been reported to influence the tumor immune microenvironment and prognosis in bladder cancer (17). Similarly, MIR516A promotes bladder cancer cell growth by attenuating autophagy. MIR516A binds to the PHLPP2 3′-UTR to inhibit PHLPP2 expression, subsequently promoting CUL4A-mediated BECN1 protein degradation to regulate autophagy (18). As a result, evasion of multiple types of programmed cell death is a hallmark of human bladder cancer. A greater understanding of Pan-PCD may contribute to mitigating tumorigenesis, tumor progression, and treatment resistance in bladder cancer.

Early studies of PCD in bladder cancer focused on specific types of cell death, such as necroptosis and pyroptosis (19). However, multiple types of programmed cell death comprehensively regulate tumorigenesis as well as tumor progression and metastasis. A systemic exploration of the role of Pan-PCD in bladder cancer is lacking. In this study, we evaluated the expression pattern of Pan-PCD in bladder cancer and conceptualized multiple types of PCD as “Pan-PCD”. Based on the novel concept of Pan-PCD, we developed a Pan-PCD-related prognostic signature to predict patient prognosis. Notably, we verified that the Pan-PCD signature can improve the accuracy of the prognostic prediction of bladder cancer.

## Material and methods

### Data source

Bladder cancer transcriptome data were downloaded from The Cancer Genome Atlas (TCGA) (https://portal.gdc.cancer.gov/). A total of 409 subjects and 19 controls were included in our study. Corresponding clinicopathological data of patients were obtained from TCGA Bladder Cancer cohort. Our Pan-PCD gene set comes from the reported multiple types of PCD-related genes, which includes 87 apoptosis genes, 51 necroptosis genes, 59 ferroptosis genes, 39 pyroptosis genes, 136 autophagy genes, and 6 cuproptosis genes (**Table 1**). We totally obtained 378 Pan-PCD-related genes. The presence of tumor-infiltrating immune cells in TCGA-BLCA samples was estimated by TIMER2.0 (http://timer.compgenomics.org/). The m6A-related gene list was obtained from a previous study (20). The IMvigor210 cohort was a multicenter, phase 2 clinical trial that evaluated the efficacy and safety of atezolizumab, an anti-PD-L1 monoclonal antibody, in metastatic urothelial carcinoma (mUC) (21). Therefore, the IMvigor210 cohort was used to validate the potential effect of PCDs on immunotherapy for bladder cancer. More cohorts, including three pathological types of kidney cancer and prostate adenocarcinoma from TCGA, as well as two independent bladder cancer cohorts ArrayExpress (E-MTAB-4321) and Gene Expression Omnibus (GSE13507), were used to validate the prognostic value of Pan-PCD signature.

**Table 1:** List of pan-PCD genes.

type	Gene number	Gene name
apoptosis	87	AIFM1 AKT1 AKT2 AKT3 APAF1 ATM BAD BAX BCL2 BCL2L1 BID BIRC2 BIRC3 CAPN1 CAPN2 CASP10 CASP3 CASP6 CASP7 CASP8 CASP9 CFLAR CHP1 CHP2 CHUK CSF2RB CYCS DFFA DFFB ENDOD1 ENDOG EXOG FADD FAS FASLG IKBKB IKBKG IL1A IL1B IL1R1 IL1RAP IL3 IL3RA IRAK1 IRAK2 IRAK3 IRAK4 MAP3K14 MYD88 NFKB1 NFKBIA NGF NTRK1 PIK3CA PIK3CB PIK3CD PIK3CG PIK3R1 PIK3R2 PIK3R3 PIK3R5 PPP3CA PPP3CB PPP3CC PPP3R1 PPP3R2 PRKACA PRKACB PRKACG PRKAR1A PRKAR1B PRKAR2A PRKAR2B PRKX RELA RIPK1 TNF TNFRSF10A TNFRSF10B TNFRSF10C TNFRSF10D TNFRSF1A TNFSF10 TP53 TRADD TRAF2 XIAP
autophagy	136	ACBD5 AKT1S1 AMBRA1 ATG10 ATG101 ATG12 ATG13 ATG14 ATG16L1 ATG16L2 ATG2A ATG2B ATG3 ATG4A ATG4B ATG4C ATG4D ATG5 ATG7 ATG9A ATG9B BCL2 BECN1 BNIP3 BNIP3L CALCOCO2 COX4I1 CTSB CTSD CTSL DEPTOR DNM1L DRAM1 DRAM2 EPG5 FIS1 FUNDC1 GABARAP GABARAPL1 GABARAPL2 HIF1A HSPD1 KIF5B LAMP1 LAMP2 MAP1LC3A MAP1LC3B MAP1LC3B2 MAP1LC3C MFN1 MFN2 MLST8 MTOR NBR1 OPTN PHB2 PIK3C3 PIK3R4 PINK1 PLEKHM1 PRKAA1 PRKN RAB11A RAB1A RAB24 RAB33B RAB5A RAB7A RB1CC1 RICTOR RPTOR RUBCN SH3GLB1 SIRT1 SNAP29 SQSTM1 STX17 TAX1BP1 TBC1D15 TBC1D17 TBC1D5 TBK1 TECPR1 TFEB TIMM23 TOMM20 TP53INP2 TSC1 TSC2 ULK1 ULK2 ULK3 USO1 USP30 UVRAG VAMP8 VDAC1 VMP1 WDFY3 WDR45 WDR45B WIPI1 WIPI2 ZFYVE1 ZKSCAN3 APP ATP6AP2 C9orf72 CLEC16A CTNS ERBB2 GBA GPR65 GRN HTT IRGM LRRK2 MECP2 MEFV MTMR3 PEX13 PSEN1 PTPN2 RETREG1 SLC33A1 SMS SMURF1 SNX14 SPG11 TCIRG1 TECPR2 TMEM230 VPS13D WAS ZFYVE26 ATG32 NRBF2
cuproptosis	6	ATP7B DLAT FDX1 GSH LIAS SLC31A1
ferroptosis	59	ABCC1 ACACA ACO1 ACSF2 ACSL3 ACSL4 AIFM2 AKR1C1 AKR1C2 AKR1C3 ALOX12 ALOX15 ALOX5 ATP5MC3 CARS CBS CD44 CHAC1 CISD1 CRYAB CS DPP4 EMC2 FADS2 FANCD2 FDFT1 FTH1 G6PD GCLC GCLM GLS2 GOT1 GPX4 GSS HMGCR HMOX1 HSBP1 HSPB1 IREB2 KEAP1 LPCAT3 MT1G NCOA4 NFE2L2 NFS1 NOX1 NQO1 PEBP1 PGD PHKG2 PTGS2 RPL8 SAT1 SLC1A5 SLC7A11 SQLE STEAP3 TFRC TP53 ZEB1
necroptosis	51	ALK APP ATRX AXL BACH2 BCL2 BCL2L11 BNIP3 BRAF CASP8 CD40 CDKN2A CFLAR CYLD DDX58 DIABLO DNMT1 EGFR FADD FAS FASLG FLT3 GATA3 HAT1 HDAC9 HSP90AA1 HSPA4 ID1 IDH1 IDH2 IPMK ITPK1 KLF9 LEF1 MAP3K7 MAPK8 MLKL MPG MYC MYCN OTULIN PANX1 PLK1 RIPK1 RIPK3 RNF31 SIRT1 SIRT2 SIRT3 SLC39A7 SPATA2 SQSTM1 STAT3 STUB1 TARDBP TERT TLR3 TNF TNFRSF1A TNFRSF1B TNFRSF21 TNFSF10 TRAF2 TRIM11 TSC1 USP22 ZBP1
pyroptosis	39	AIM2 BAK1 BAX CASP1 CASP3 CASP4 CASP5 CASP6 CASP8 CASP9 CHMP2A CHMP2B CHMP3 CHMP4A CHMP4B CHMP4C CHMP6 CHMP7 CYCS ELANE GPX4 GSDMA GSDMB GSDMC GSDMD GSDME GZMB HMGB1 IL18 IL1A IL1B IL6 IRF1 IRF2 NLRC4 NLRP1 NLRP2 NLRP3 NLRP6 NLRP7 NOD1 NOD2 PJVK PLCG1 PRKACA PYCARD SCAF11 TIRAP TNF TP53 TP63

### Identification of differentially expressed PCD genes

The expression matrix of Pan-PCD-related genes was extracted. Then, the differentially expressed genes (DEGs) between tumor and control specimens were screened using the “edgeR” R package. Here, log2|fold change>1| and an adjusted p value (padj) <0.05 were established as the cutoff values. The R package “ggplot2” was used to visualize the volcano plots and box plots of DEGs. Heatmaps were generated using the R package “pheatmap”.

### Functional analysis of DEGs

Gene Ontology (GO) and Kyoto Encyclopedia of Genes and Genomes (KEGG) analyses were conducted to explore potential molecular processes and biological pathways related to Pan-PCD in bladder cancer. These analyses were performed using the R package “clusterProfiler” for all Pan-PCD-related DEGs. The p values < 0.05 were considered statistically significant.

### Pan-PCD-related prognostic signature evaluation

The prognostic value of PCD genes in the TCGA-BLCA cohort was investigated by univariate Cox regression analysis. Multivariate Cox regression analysis was subsequently performed to identify independent prognosis-related genes, and we constructed the Pan-PCD-related prognostic signature. A Kaplan–Meier plot of survival curves was utilized to evaluate the survival probability of the low- and high-PPRPS groups. Using the “timeROC”, “survival”, and “survminer” R packages, the time-dependent receiver operating characteristic curve (ROC) curve was drawn based on the calculated PPRPS score. The area under the curve (AUC) represents the 1-, 2-, and 3-year overall survival (OS) probability to estimate the accuracy of the predicted survival probability and the actual observation rate.

### Independent prognostic analysis of PPRPS

We obtained the clinical parameters of patients from TCGA-BLCA cohort. These clinical variables and PPRPS were combined and analyzed in the regression model. Univariate and multivariate Cox regression models were utilized to investigate independent prognostic factors. Forest plots were used to show the results of univariate and multivariate Cox regression analyses using the “forestplot” R package.

### Nomogram development and decision curve analysis

A nomogram was generated to visualize PPRPS and for use in clinical applications. To evaluate the value of clinical characteristics in predicting the OS probability of bladder cancer patients, we developed multiple decision curves based on the clinical characteristics, PPRPS, and combined clinical-PPRPS models. The clinical application could be determined by quantifying the net benefits at different threshold probabilities.

### Gene set enrichment analysis

To further explore the difference in the Pan-PCD-related prognostic signature, gene set enrichment analysis (GSEA) was performed to investigate the Pan-PCD-related prognostic signature with P values < 0.05 indicating enrichment for significant functional annotation. The detailed parameters were described in a previous study (22). The annotated gene sets of C2,cp.kegg.v7.0.symbols.gmt in the Molecular Signatures Database (MSigDB) version 7.5.1 were selected in GSEA version 4.2.3. The number of permutations was 1,000. The collapse dataset with gene symbols was set as “False.” The permutation type was set as “phenotype.” GSEA was performed. The cutoff criteria were as follows: false discovery rate (FDR) q > 0.25, nominal p < 0.05, and normalized enrichment scores (NES) > 1.5.

### Immune landscape in low- and high-PPRPS bladder cancer patients

The difference in tumor-infiltrating immune cells between the two PPRPS groups was estimated based on the following 7 algorithms: CIBERSORT, TIMER, CIBERSORE-ABS, MCPCOUNTER, QUANTISEQ, XCELL and EPIC. In addition, the characteristics of the tumor microenvironment were also evaluated through scoring with the “ssGSEA” method. Furthermore, a few checkpoint molecules were analyzed to investigate the differences between the low- and high-PPRPS groups.

### Identification of differentially expressed m6A-related genes

m6A modification regulates the expression level of PCD genes. We selected 12 m6A RNA methylation regulators, including 2 erasers (ALKBH5 and FTO), 5 writers (METTL3, METTL14, WTAP, RBM15, and ZC3H13), and 5 readers (HNRNPC, YTHDC1, YTHDC2, YTHDF1, and YTHDF2). To identify the expression differences in m6A regulators between the low- and high-PPRPS groups, a *t*-test was used.

### Evaluation of the effect of pan-PCDs on anti-PD-L1 immunotherapy in bladder cancer

The IMvigor210 cohort was used to assess the effect of anti-PD-L1 immunotherapy. The best overall response was defined as the best response recorded from the start of the treatment until disease progression/relapse. The best overall response was summarized by response category as follows: complete response (CR), partial response (PR), stable disease (SD), or progressive disease (PD). The Kaplan–Meier method was used to estimate OS rates.

### Chemical response prediction

Based on the pharmacogenomics database Genomics of Drug Sensitivity in Cancer (GDSC) (https://www.cancerrxgene.org/), we predicted the chemotherapy response of each sample. Twenty anticancer drugs effective against bladder cancer were selected. The analysis was performed using the R package “oncoPredict”. The IC_50_ of each sample was estimated, and the different IC_50_ values between the high- and low-PPRPS groups were statistically analyzed using the Wilcoxon method.

## Results

### Identification of differentially expressed pan-PCD-related genes in bladder cancer

**Figure 1:**
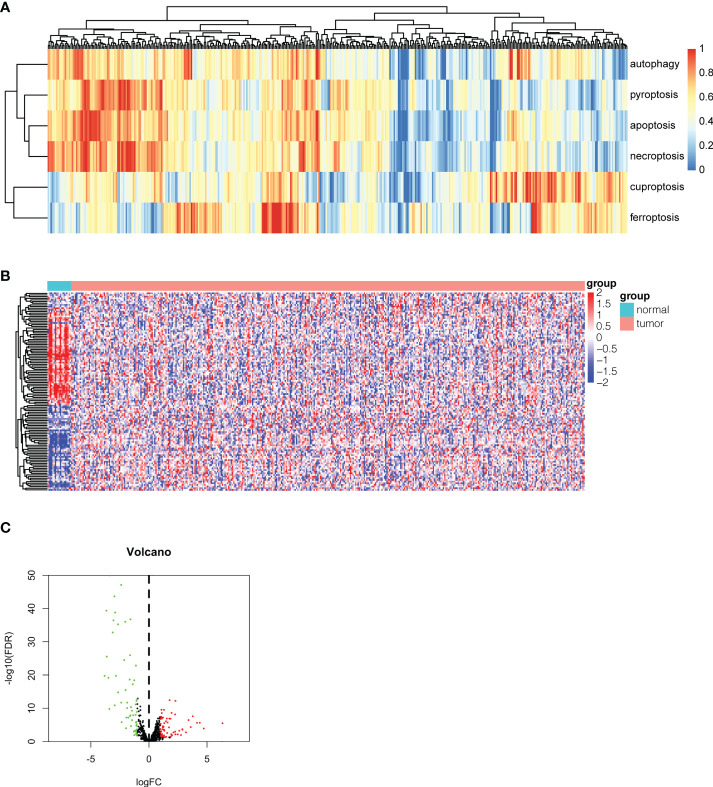
Identification of differentially expressed Pan-PCD-related genes in bladder cancer. **(A)** Heatmap of ssGSEA showing the enrichment scores of the Pan-PCD gene set in each bladder cancer tissue. **(B)** The heatmap revealed the expression level of 378 Pan-PCD-related genes between tumor and normal tissues. **(C)** The volcano plot displays Pan-PCD DEGs discovered in tumor tissue. Sixty-one genes showed significantly higher expression (red dots), whereas 59 genes showed significantly lower expression (green dots) in bladder cancer.

To investigate the expression pattern of different PCD biological behaviors in bladder cancer, single-sample gene set enrichment analysis (ssGSEA) was conducted to assess the enrichment scores of our Pan-PCD gene set in every sample we chose. The ssGSEA results are shown in **Figure 1A**. The heatmap revealed the differences in each sample among the six types of PCDs, and these samples were subclustered according to the expression pattern of Pan-PCD enrichment scores. To identify the potential Pan-PCD-related DEGs specific to bladder cancer, the expression levels of 378 Pan-PCD-related genes were compared between tumor and normal tissues obtained from bladder cancer patients (**Figure 1B**). Among the comparisons, a total of 120 Pan-PCD-related DEGs were discovered in tumor tissue. Sixty-one Pan-PCD-related genes showed significantly higher expression, whereas 59 Pan-PCD-related genes revealed significantly decreased expression in bladder cancer (**Figure 1C**). We subsequently used these Pan-PCD-related DEGs to perform further analysis.

### GO enrichment and KEGG pathway analysis of the pan-PCD-related DEGs

**Figure 2:**
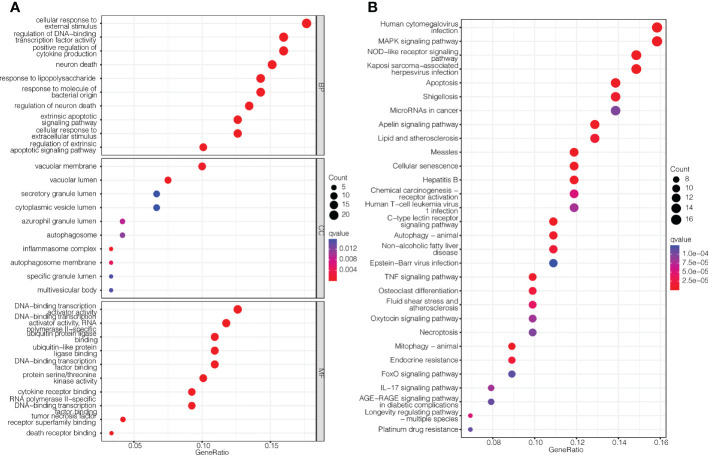
GO enrichment and KEGG pathway analysis of the Pan-PCD-related DEGs. **(A)** Top 10 terms for each GO category of the Pan-PCD-related DEGs. **(B)** Top 30 enriched KEGG pathways of the Pan-PCD DEGs.

To investigate the biological functions and relevant pathways of these DEGs, GO and KEGG analyses were performed. The top 10 terms for each GO category and the top 30 pathways for KEGG are shown in **Figure 2**. The biological processes of the Pan-PCD-related DEGs involved in the GO analysis included cellular response to external stimulus, regulation of DNA-binding transcription factor activity, and positive regulation of cytokine production. The cellular components of the Pan-PCD-related DEGs of GO were mainly vacuolar membrane, vacuolar lumen, and secretory granule lumen. The molecular functions of the Pan-PCD-related DEGs of GO included DNA-binding transcription activator activity, ubiquitin protein ligase binding, ubiquitin-like protein ligase binding, and others. Additionally, the DEGs enriched in the KEGG pathway were largely related to the MAPK signaling pathway, NOD-like receptor signaling pathway, microRNAs in cancer, and the apelin signaling pathway. In addition, some Pan-PCD-related pathways also revealed significant enrichment in KEGG, including apoptosis, autophagy-animal, and necroptosis.

### Construction of a pan-PCD-related prognostic signature in bladder cancer

**Figure 3:**
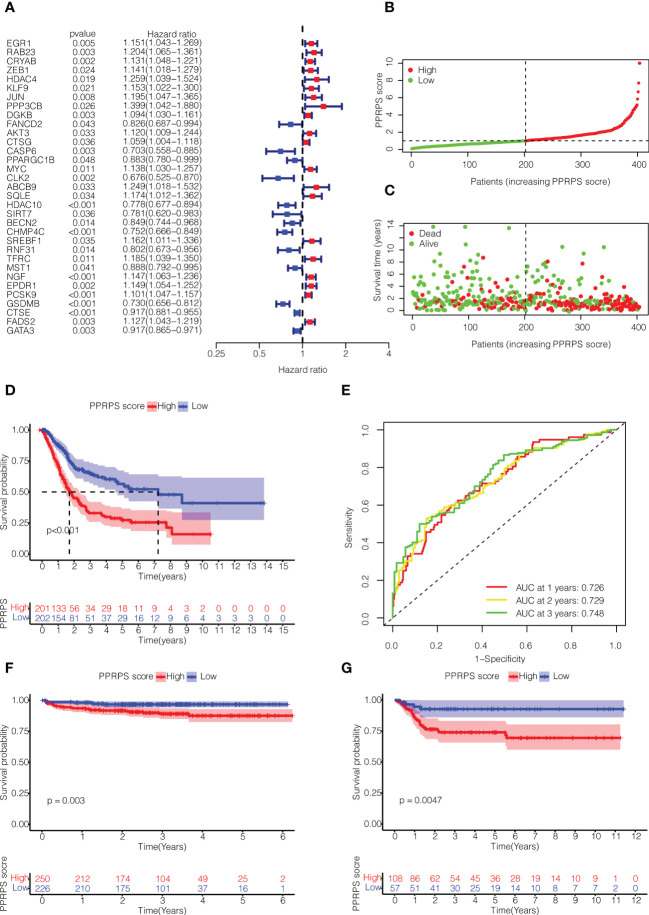
Construction of the Pan-PCD-related prognostic signature in bladder cancer. **(A)** Univariate Cox regression analysis of Pan-PCD-related DEGs. **(B)** Distribution of bladder cancer patient survival based on TCGA cohort data. **(C)** The distributions of status PPRPS showed that the number of deaths increased as the PPRPS increased. **(D)** Kaplan–Meier (K-M) curves of the high-PPRPS and low-PPRPS groups. Patients in the high-PPRPS group had a poor prognosis compared to the low-PPRPS group (p<0.001). **(E)** AUC of time-dependent ROC curves. **(F)** K-M curves of the high- and low-PPRPS groups in ArrayExpress cohort E-MTAB-4321. **(G)** K-M curves of the high- and low-PPRPS groups in GEO cohort GSE13507.

Based on the differentially expressed Pan-PCD-related genes, 33 Pan-PCD-related genes with prognostic value were preliminarily identified *via* univariate Cox regression analysis with p<0.05 (**Figure 3A**). Among them, 20 genes with hazard ratio (HR) > 1 were related to increased risk, whereas the remaining 13 genes with HR < 1 were protective genes. Then, multivariate Cox regression analysis was performed, and 15 genes were used to construct a Pan-PCD-related prognostic signature in bladder cancer as noted in the following formula: EGR1*0.091+KLF9*(-0.241) + PPP3CB*0.236+FANCD2*(-0.346) +MYC*0.102+ABCB9*0.343+HDAC10*(-0.120) + BECN2*(-0.127) + CHMP4C*(-0.130) + SREBF1*0.169 +TFRC*0.178 + NGF*0.090 + EPDR1*0.115 + CTSE*(-0.045) + FADS2*0.139.

Then, these patients were divided into low-PPRPS and high-PPRPS subgroups according to the median PPRPS score as the cutoff value (**Figure 3B**). As shown in **Figures 3C, D**, the number of deaths was increased in the high-PPRPS group compared to the low-PPRPS group, and higher PPRPS were associated with significantly shorter survival times (P < 0.001), as shown in **Figure 3D**. Finally, to evaluate the specificity and sensitivity of the Pan-PCD prognostic model, 1-, 3-, and 5-year receiver operating curves (ROCs) were constructed, and the area under the ROC curve (AUC) was 0.726 at 1 year, 0.729 at 2 years, and 0.748 at 3 years (**Figure 3E**).

We further validated the prognostic value of PPRPS in two other independent bladder cancer cohorts from ArrayExpress (E-MTAB-4321) and Gene Expression Omnibus (GSE13507). As shown in **Figures 3F, G**, a higher PPRPS score was associated with significantly lower survival probabilities in both of these two cohorts (P = 0.003 and P = 0.0047, respectively), which is quite in accordance with our prognostic signature.

### Evaluation of the independent prognostic value of pan-PCD-related prognostic models

**Figure 4:**
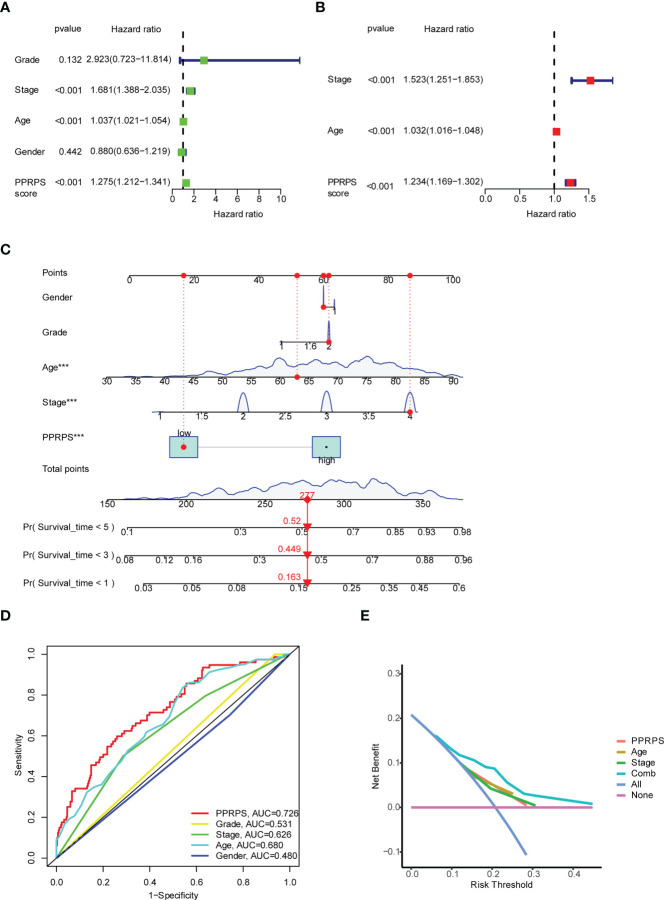
Univariate and multivariate Cox analyses of the PPRPS and clinical characteristics based on Pan-PCD signature genes. **(A, B)** Predicting overall survival (OS) based on univariate **(A)** and multivariate **(B)** Cox analyses. **(C)** Nomogram to predict the 1-, 3- and 5-year OS of bladder cancer patients. **(D)** Comparison of 1-year ROC curves with clinicopathological features. **(E)** Decision curve analysis (DCA) of the clinicopathological model predicts the net increase in the risk of bladder cancer.

To further validate the clinical application of our Pan-PCD-related prediction model, we integrated the patients’ clinical information, including grade, stage, age, and PPRPS, to perform univariate and multivariate Cox analyses to develop the nomogram for OS (overall survival). The results in **Figures 4A, B** show that stage, age, and the PPRPS were independent prognostic predictors of OS. The nomogram predicts the 1-year, 3-year, and 5-year OS of bladder cancer patients (**Figure 4C**). Moreover, the 1-year ROC curves with clinicopathological features, including grade, stage, age, sex, and PPRPS, are shown in **Figure 4D**. The AUCs were 0.726 for the PPRPS, 0.680 for age, and 0.626 for stage. The decision curve analysis (DCA) of different models is shown in **Figure 4E**. The results indicated relatively acceptable concordance between the predicted probabilities of the survival model and the actual bladder cancer recurrence within 1 year after surgery. The DCA results reveal a large distance between the combination curve and all other curves, suggesting that the combination of PPRPS, age, and stage was the key index to evaluate the prognosis.

### Clustering of tumor samples based on pan-PCD DEGs and functional analysis of pan-PCD DEGs in the prognostic model

**Figure 5:**
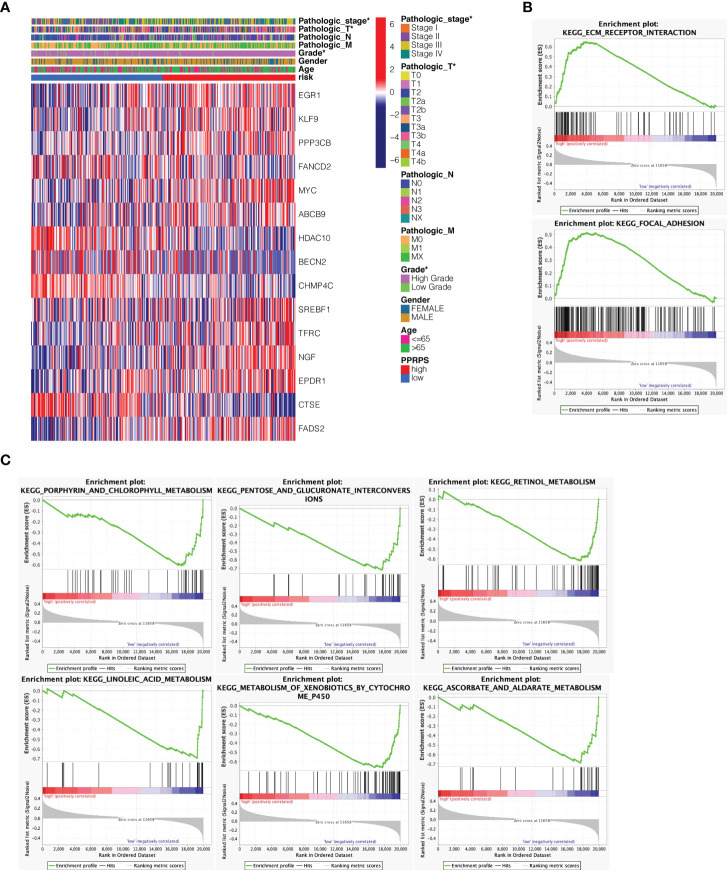
Clustering of tumor samples based on Pan-PCD DEGs and functional analysis of the prognostic model. **(A)** Relevance between the PPRPS and clinical features among 16 DEGs. The pathologic stage, T stage and pathological grade differed between the low- and high-PPRPS subgroups (P < 0.05). **(B)** Pathways enriched in the high-PPRPS group. **(C)** Pathways enriched in the low-PPRPS group.

The heatmap of 15 Pan-PCD genes in the Pan-PCD-related prognostic signature is displayed in **Figure 5A**, and the clinical characteristics were analyzed. The distribution heatmap revealed significant differences in pathologic stage, T stage, and pathological grade between the low- and high-PPRPS subgroups (p < 0.05). To further explore the functional differences in the biological pathways, GSEA of transcriptome patterns between prognostic models was performed. As shown in **Table 2** and **Figure 5B, C**, focal adhesion, ECM receptor interaction, and other pathways were enriched in the high-PPRPS group, whereas porphyrin and chlorophyll metabolism, pentose and glucuronate interconversions, linoleic acid metabolism, metabolism of xenobiotics by cytochrome P450, and retinol metabolism signaling pathways were enriched in the low-PPRPS group. The results indicated that the pathways related to immune factors, cellular adhesion, and tumor metastasis were involved in the high-PPRPS group of bladder cancer, whereas altered metabolic status played a major role in the low-PPRPS group, which was tightly associated with different PCD classifications.

**Table 2:** KEGG signaling pathways enriched in different PPRPS groups.

KEGG signaling pathways enriched in the low-risk group					
NAME	SIZE	ES	NES	NOM p-val	FDR q-val
KEGG_LINOLEIC_ACID_METABOLISM	29	-0.695	-1.584	0.008	0.418
KEGG_PENTOSE_AND_GLUCURONATE_INTERCONVERSIONS	28	-0.724	-1.625	0.018	0.461
KEGG_METABOLISM_OF_XENOBIOTICS_BY_CYTOCHROME_P450	68	-0.665	-1.565	0.018	0.365
KEGG_PORPHYRIN_AND_CHLOROPHYLL_METABOLISM	41	-0.609	-1.640	0.027	0.813
KEGG_RETINOL_METABOLISM	64	-0.621	-1.538	0.030	0.351
KEGG_ASCORBATE_AND_ALDARATE_METABOLISM	25	-0.691	-1.517	0.035	0.341
KEGG signaling pathways enriched in the high-risk group					
NAME	SIZE	ES	NES	NOM p-val	FDR q-val
KEGG_NICOTINATE_AND_NICOTINAMIDE_METABOLISM	24	0.681	1.755	0.002	0.707
KEGG_ECM_RECEPTOR_INTERACTION	84	0.647	1.659	0.009	0.574
KEGG_GLIOMA	65	0.475	1.621	0.015	0.591
KEGG_FOCAL_ADHESION	199	0.512	1.620	0.038	0.479
KEGG_REGULATION_OF_ACTIN_CYTOSKELETON	212	0.457	1.593	0.014	0.429
KEGG_PRION_DISEASES	35	0.573	1.565	0.032	0.415
KEGG_SMALL_CELL_LUNG_CANCER	84	0.470	1.562	0.036	0.383
KEGG_SNARE_INTERACTIONS_IN_VESICULAR_TRANSPORT	38	0.433	1.553	0.041	0.343
KEGG_PANTOTHENATE_AND_COA_BIOSYNTHESIS	16	0.645	1.551	0.034	0.320
KEGG_HEMATOPOIETIC_CELL_LINEAGE	85	0.681	1.541	0.043	0.317
KEGG_BIOSYNTHESIS_OF_UNSATURATED_FATTY_ACIDS	22	0.566	1.541	0.046	0.297
KEGG_OOCYTE_MEIOSIS	112	0.375	1.517	0.048	0.328

PPRPS, Pan-PCD-related prognostic signature.

### Immune landscape between low- and high-PPRPS bladder cancer patients

**Figure 6:**
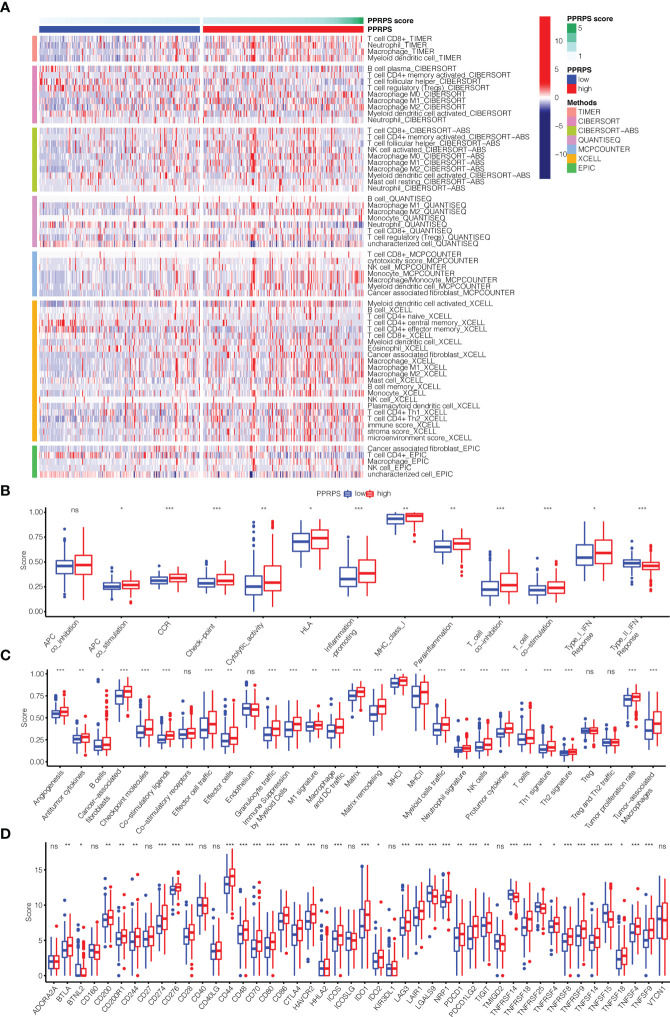
Immune microenvironment, immune-related pathways, tumor microenvironment (TME) and checkpoint genes in the low- and high-PPRPS groups. **(A)** Heatmap of the immune microenvironment revealing the immune cells and stromal score between the two PPRPS groups. **(B)** Scores of 13 immune-related pathways between the two PPRPS groups. **(C)** Twenty-nine functional gene expression signatures (Fges) describing the TME in the two PPRPS groups. **(D)** Expression of immune checkpoint genes in the high- and low-PPRPS groups. *p < 0.05, **p < 0.01, ***p < 0.001, ns, not significant.

The differences in immune cell infiltration between the two PPRPS groups were calculated based on the 7 algorithms. The results shown in **Figure 6A** suggested that many tumor-infiltrating immune cells displayed significant differences between the low- and high- PPRPS groups, and most of these cells were enriched in the high-PPRPS group. According to the different algorithms, 65 types of immune cells were significantly different between the two PPRPS groups. The detailed immune cell scores of each type of immune cell based on 7 algorithms are summarized in **Supplementary Figure 1**. For example, according to the results of CIBERSORT, CIBERSORE-ABS, QUANTISEQ, and XCELL, both M1 and M2 macrophages were significantly increased in the high-PPRPS group. TIMER, CIBERSORE-ABS, QUANTISEQ, XCELL, and EPIC results reveal that CD8^+^ T cells were significantly increased in the high-PPRPS group. However, activated myeloid dendritic cells exhibited a significant decrease in the high-PPRPS group based on CIBERSORT, CIBERSORE-ABS, XCELL, and MCPCOUNTER results. We further analyzed 13 immune-related signatures (**Figure 6B**), and most of these signatures were significantly upregulated in the high-PPRPS group compared with the low-PPRPS group. Only the Type_II_IFN_Response showed significant downregulation in the high-PPRPS group, whereas no difference in APC_co_inhibition was observed. To explore the possible role of the tumor microenvironment (TME), the expression signature of 29 TME-related genes (23) is illustrated in **Figure 6C** (23). Most TME signatures exhibited increased levels, indicating increased biological activity in the high-PPRPS group. Because immune checkpoints play vital roles in cancer treatment, the expression of checkpoints was compared between the two PPRPS groups (**Figure 6D**). We found that only two genes, TNFRSF14 and TNFSF15, exhibited increased expression in the low-PPRPS group, whereas most immune checkpoint-related genes were highly expressed in the high-PPRPS group. For example, immune checkpoints, including CD274 (PD-L1), PDCD1 (PD-1), and PDCD1LG2 (PD-L2), exhibited increased expression in the high-PPRPS group, revealing immunotherapy as a potential treatment for high-PPRPS patients.

### Identification of DEGs of m6A patterns between low- and high-PPRPS bladder cancer patients

**Figure 7:**
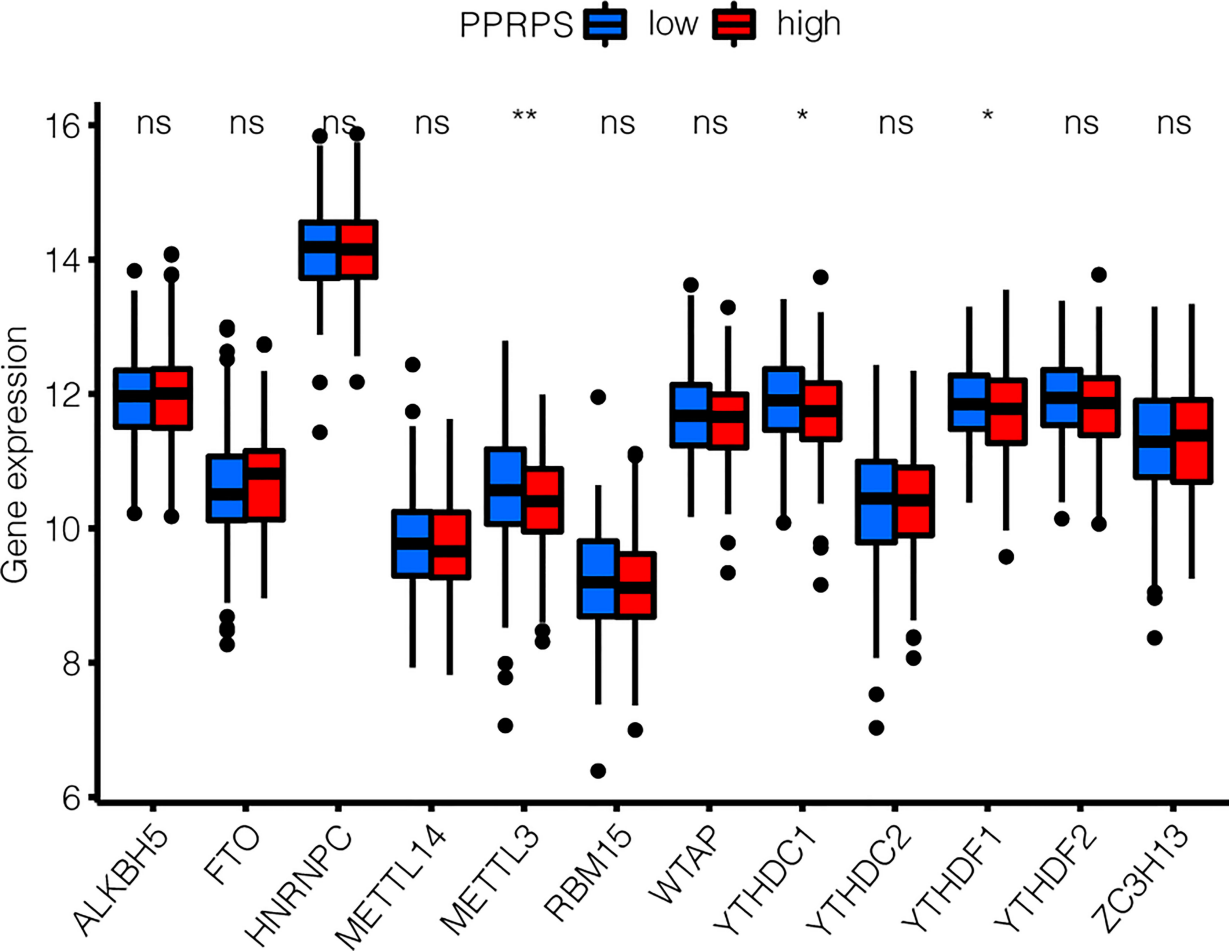
m6A-related DEG patterns between low- and high-PPRPS bladder cancer patients. The expression of 12 m6A-related genes was evaluated within the low- and high-PPRPS groups. *p < 0.05, **p < 0.01, ns, not significant.

Recent studies have shown that aberrant m6A methylation and related regulatory proteins are closely associated with a variety of cancers (24), including bladder cancer (25). Therefore, the m6A-related DEGs were analyzed in the low- and high-PPRPS groups. Increased METTL3, YTHDC1, and YTHDF1 expression were noted in the low-PPRPS group than in the high-PPRPS group (**Figure 7**).

### Effect of PPRPS on anti-PD-L1 immunotherapy in pan-PCD bladder cancer subtypes

**Figure 8:**
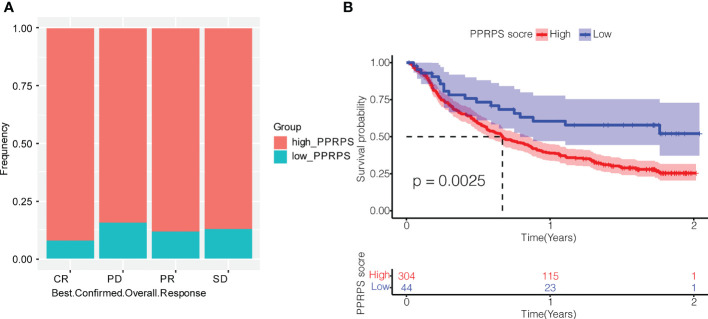
Effect of Pan-PCDs on anti-PD-L1 immunotherapy for bladder cancer. **(A)** Evaluation of the best overall response. Confirmation of CR, PD, PR, and SD in the low- and high-PPRPS groups. CR, complete response; PR, partial response; SD, stable disease; PD, progressive disease. **(B)** Kaplan–Meier (K-M) curve of the high-PPRPS and low-PPRPS groups after anti-PD-L1 immunotherapy, demonstrating that the patients in the high-PPRPS group had a poorer prognosis than those in the low-PPRPS group (p = 0.0025).

To explore the potential effect of PPRPS on immunotherapy for bladder cancer, we obtained data from the IMvigor210 cohort to evaluate the efficacy of atezolizumab, an anti-PD-L1 monoclonal antibody, in patients with metastatic urothelial cancer. The frequency of high-PPRPS patients in different groups of immune responses is presented in **Figure 8A**. The number of deaths was greater in the high-PPRPS group compared with the low-PPRPS group (p = 0.0025). The results indicated that the Pan-PCD-related prognostic signature exerts very few impacts on anti-PD-L1 immunotherapy. The low-PPRPS group showed a better therapeutic effect after treatment, but the high-PPRPS group showed a limited effect (**Figure 8B**).

To further explore the immune landscape between the low- and high-PPRPS groups, 13 immune-related signatures, 29 TME-related gene expression signatures, and gene expression of checkpoints were analyzed. As shown in **Supplementary Figure 2A**, all immune-related signatures were significantly upregulated in the high-PPRPS group compared with the low-PPRPS group with the exception of type_II_IFN_Response, which showed no difference between the two groups. The 29 TME-related gene expression signatures (23) illustrated in Supplementary **Figure 2B** (23) revealed that most TME signatures exhibited increased expression in the high-PPRPS group, whereas only the tumor proliferation rate was significantly downregulated. Moreover, the scores of checkpoint-related genes were compared between the two PPRPS groups (**Supplementary Figure 2C**). Most immune checkpoint-related genes demonstrated higher scores in the high-PPRPS group, including CD274 (PD-L1), PDCD1 (PD-1), and PDCD1LG2 (PD-L2). Taken together, these immune landscape characteristics strongly support our therapeutic finding that the low PPRPS group benefits more from anti-PD-L1 immunotherapy.

### Sensitivity of pan-PCD subtypes to chemotherapy

**Figure 9:**
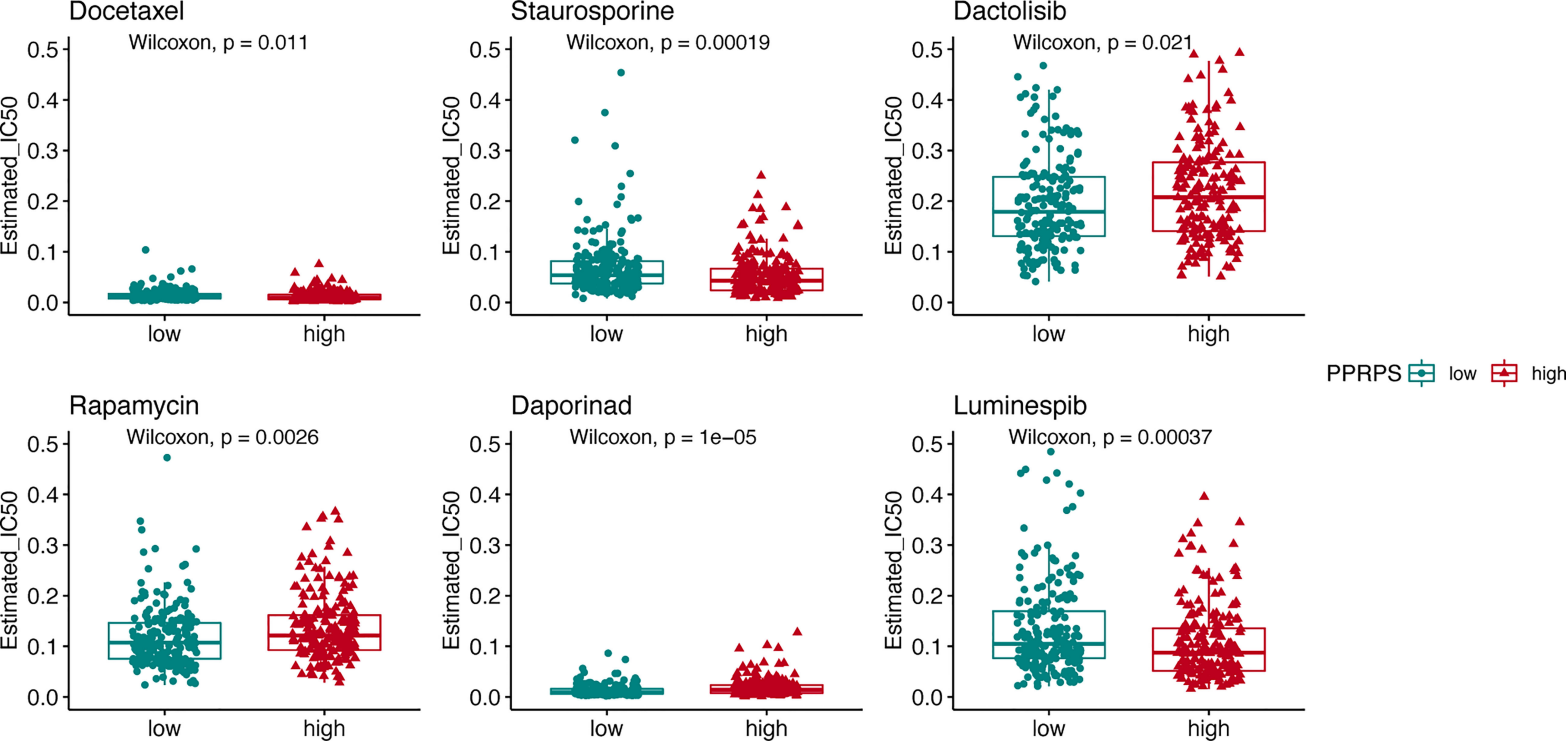
The pan-PCD score of chemotherapeutic agents during the courses of cancer chemotherapy. The IC_50_ values of chemotherapeutic agents among the high- and low-PPRPS groups are shown in the figure.

Given that the high-PPRPS group did not show sensitivity to anti-PD-1 treatment, we further aimed to determine whether conventional chemotherapy would exhibit efficacy in this group of patients. In total, 20 chemotherapy drugs were selected, and the responses of the low- and high-PPRPS groups to these drugs were evaluated based on the IC_50_ of each sample in our TCGA dataset. According to **Figure 9**, the anticancer drugs docetaxel, staurosporine, dactolisib, rapamycin, daporinad, and luminespib exhibited significantly different effects between different pan-PCD subtypes. Among them, higher sensitivity to docetaxel, staurosporine, and luminespib was noted the high-PPRPS group (p= 0.011, p= 0.00019, and p= 0.00037). However, patients in the high-PPRPS group were less sensitive to dactolisib, rapamycin, and daporinad compared with the low-PPRPS group (p= 0.021, p= 0.0026, and p= 1e−05). Together, our results suggested that docetaxel, staurosporine, and luminespib are effective in high-PPRPS bladder cancer treatment.

### Prognostic value of PPRPS for kidney cancer and prostate cancer

To further investigate the prognostic value of PPRPS in genitourinary cancers, we chose another three pathological types of kidney cancer and prostate cancer for validation. The results indicated that PPRPS has a good prognostic value for kidney renal clear cell carcinoma (KIRC), kidney renal papillary cell carcinoma (KIRP), and prostate adenocarcinoma (PRAD), but not for kidney chromophobe (KICH) in **Supplementary Figure 3**. In contrast with the results of bladder cancer, KIRC, and KIRP, the patients with high PPRPS had a good prognosis, suggesting a different Pan-PCD expression pattern in PRAD.

## Discussion

Cell death has been established as an important anticancer defense mechanism and therapeutic target. Evasion of malignant cells from cell death is regarded as a crucial requirement for malignant transformation and tumorigenesis. In this sense, exploring the mechanisms and functions of cell death, especially the forms of programmed cell death and the steps involved in regulated cell deaths, would shed some light on tumor development and anticancer therapy. Bladder cancer has shown many different PCD-associated signatures that can be used to understand death resistance (26), prognosis (27), the tumor microenvironment (17), and other features of bladder cancer. In our present study, we aimed to answer three questions: First, can Pan-PCD be applied as a signature to bladder cancer subtype classification given that various PCDs serve as a defense mechanism against oncogenesis as well as the tumor microenvironment in bladder cancer? Second, can bladder cancer subtypes be used to predict the prognosis and immune infiltration of specific bladder cancer patients? Finally, are the new pan-PCD molecular subtypes associated with the response to anticancer therapy?

In our study, we utilized Pan-PCD-related DEGs to construct a prognostic signature in bladder cancer. We discovered that bladder cancer exhibited varied Pan-PCD levels based on our TCGA datasets, and these levels were used to create the high-PPRPS group and the low-PPRPS group. The bladder cancer subtypes revealed an intrinsic cell death signature in patients, which subsequently impacted patient prognosis. Accordingly, the high-PPRPS group had a shorter survival time. The Pan-PCD-related prognostic model showed that stage, age, and the PPRPS score were independent prognostic predictors of OS. Specifically, these features were highly associated with a high PPRPS score of Pan-PCD in patients.

DEG functional analysis provides some cues on the mechanisms that characterize the high- and low-PPRPS subgroups. GSEA results in subgroups suggested that the expression of genes in the high-PPRPS group is enriched in some classical tumorigenesis-, development- and progression-related pathways. For example, focal adhesion signaling-related genes were enriched in our high-PPRPS group. The signaling in this pathway involves a wide range of prosurvival signaling molecules, including integrins, growth factor receptors, and intracellular molecules, that regulate cell behavior and tumor cell survival (28). ECMs, such as N- and P-cadherin, promote invasive and malignant phenotypes of bladder cancer (29), as evidenced by the GSEA-enriched pathways associated with the high-PPRPS group. On the other hand, dysregulated expression of some cancer-related metabolic genes was recurrently observed in the low-PPRPS group. For instance, a pan-cancer study on metabolic gene expression found that pentose and glucuronate interconversions are significantly dysregulated in a variety of cancers (30). Similarly, our results indicated that pentose and glucuronate interconversions were enriched in the low-PPRPS group. In the low-PPRPS group, retinol metabolism-related genes were also enriched. Similar findings are observed in some population-based epidemiological studies on vitamin A (including retinol, retinal, RA, and retinyl esters). A meta-analysis investigating the quantitative effects of vitamin A on bladder cancer suggested that high vitamin A intake and high blood retinol levels were associated with a reduced risk of bladder cancer (31). Moreover, retinoid therapy for cancer chemoprevention and treatment has yielded successful results in skin cancers, neuroblastomas, breast cancer, and some leukemias (32). As a result, divergent expression patterns between the high- and low-PPRPS groups may contribute to the different prognoses and survival times.

m6A modifications are increasingly recognized as an important layer of posttranscriptional gene expression regulation. Its dysregulation has been broadly reported within various cancer cells and tumors (33). Due to the critical role of m6A in controlling oncogene expression, m6A has become a promising therapeutic target for anticancer drug development (34). In our research, we identified m6A-related DEGs between the low- and high-PPRPS groups. *METTL3*, *YTHDC1*, and *YTHDF1* expressions were decreased in the high-PPRPS group, and these genes could potentially serve as candidate drug targets based on pan-PCD subtypes.

Low- and high-PPRPS bladder cancer demonstrated diverse immune landscapes, including immune cell infiltration, immune-related signatures, the tumor microenvironment, and immune checkpoints, based on our analysis. As a result, we hope to identify ideal immunotherapy for high-PPRPS bladder cancer subtypes. We further used the IMvigor210 cohort, a multicenter, 2-cohort, phase 2 trial that investigated the efficacy and safety of atezolizumab in metastatic urothelial carcinoma (mUC), to validate the potential effect of PCDs on bladder cancer. However, the high-PPRPS group showed fewer curative effects than the low-PPRPS group when anti-PD-L1 monoclonal antibody therapy was applied, indicating that patients with these high-PPRPS features will likely not benefit from anti-PD-L1 immunotherapy.

In the context of cancer treatment, many chemotherapies induce cancer cell death, including apoptosis, ferroptosis, autophagy, and pyroptosis (35–37). Hence, we focused on chemotherapy to identify chemicals to which patients in the high-PPRPS group were sensitive. The high-PPRPS group showed lower IC_50_ values for docetaxel, staurosporine, and luminespib. However, patients in the high-PPRPS group were less sensitive to dactolisib, rapamycin, and daporinad. The results may be helpful to guide subsequent therapy for individual patients. The mechanisms by which these three candidate drugs induce Pan-PCD-resistant cells in the high-PPRPS group require further study. One limitation of our study is that our results provide only some clues on the role of PPRPS in bladder cancer. However, the biological functions of Pan-PCD-related DEGs remain unclear, and further *in-vitro* or *in-vivo* experiments are required.

## Conclusion

We successfully developed a Pan-PCD-related prognostic signature of bladder cancer to predict a patient’s prognosis and perform risk prediction, and have validated the prognostic model in two other bladder cancer cohorts. Furthermore, the low- and high-PPRPS groups exhibited unique immune landscapes and m6A patterns. Finally, the low-PPRPS group benefited more from anti-PD-L1 immunotherapy, and Pan-PCD subtypes showed diverse sensitivities to chemotherapy. In conclusion, our results demonstrated that the PPRPS facilitates accurate prediction of bladder cancer prognosis and provides a novel classification method to guide the selection of therapeutic treatment.

## Data availability statement

The original contributions presented in the study are included in the article/**Supplementary Material**. Further inquiries can be directed to the corresponding author.

## Author contributions

Conceptualization, MP; Methodology, LZ, MP; Supervision, MP; Validation, LZ, MP; Writing - original draft, LZ; Writing - review & editing, LZ, MP. Both authors contributed to the article and approved the submitted version.

## Funding

This study was supported by the Natural Science Foundation of Hunan Provincial (2022JJ30831, 2021JJ40892) and the Natural Science Foundation of Changsha City (kq2202389).

## Acknowledgments

We appreciated the TCGA, GDSC, and IMVigor210 projects. We also thank American Journal Experts (AJE) for English editing.

## Conflict of interest

The authors declare that the research was conducted in the absence of any commercial or financial relationships that could be construed as a potential conflict of interest.

## Publisher’s note

All claims expressed in this article are solely those of the authors and do not necessarily represent those of their affiliated organizations, or those of the publisher, the editors and the reviewers. Any product that may be evaluated in this article, or claim that may be made by its manufacturer, is not guaranteed or endorsed by the publisher.

## Supplementary material

Supplementary Figure 1 Summary of immune cell scores of each type of immune cell between high- and low-PPRPS groups based on 7 algorithms. *p < 0.05, **p < 0.01, ***p < 0.001.

Supplementary Figure 2 Immune microenvironment, immune-related pathways, tumor microenvironment (TME) and checkpoint genes in the low- and high-PPRPS groups of the IMvigor210 cohort. **(A)** Scores of 13 immune-related pathways in the low- and high-PPRPS groups. **(B)** Twenty-nine functional gene expression signatures describing the TME in the low- and high-PPRPS groups. **(C)** Scores of immune checkpoint genes in the low- and high-PPRPS groups. *p < 0.05, **p < 0.01, ***p < 0.001.

Supplementary Figure 3 Validation of the Pan-PCDs’ effect on the prognostic value of PPRPS in kidney cancer and prostate cancer. Kaplan–Meier (K-M) curve of the high- and low-PPRPS groups in kidney renal clear cell carcinoma (KIRC) **(A)**, kidney renal papillary cell carcinoma (KIRP) **(B)**, and prostate adenocarcinoma (PRAD) **(C)** demonstrating a great prognosis in patients. **(D)** the PPRPS did not show a good prognostic value to kidney chromophobe (KICH).

## Abbreviations

TCGA, The Cancer Genome Atlas; GDSC, Genomics of Drug Sensitivity in Cancer; ssGSEA, single sample Gene Set Enrichment Analysis; K-M, Kaplan-Meier; GO, Gene Ontology; KEGG, Kyoto encyclopedia of genes and genomes; IC50, half-maximal inhibitory concentration; DEG, differentially expressed gene; ROC, receiver operating characteristic.
