# Supplementary figures and images for: Integrated bioinformatic analysis identified a novel prognostic pan-programmed cell death signature for bladder cancer
Source: Front Immunol. 2022 Nov 23;13:1030097. doi: 10.3389/fimmu.2022.1030097 (PMC9728529; doi:10.3389/fimmu.2022.1030097)

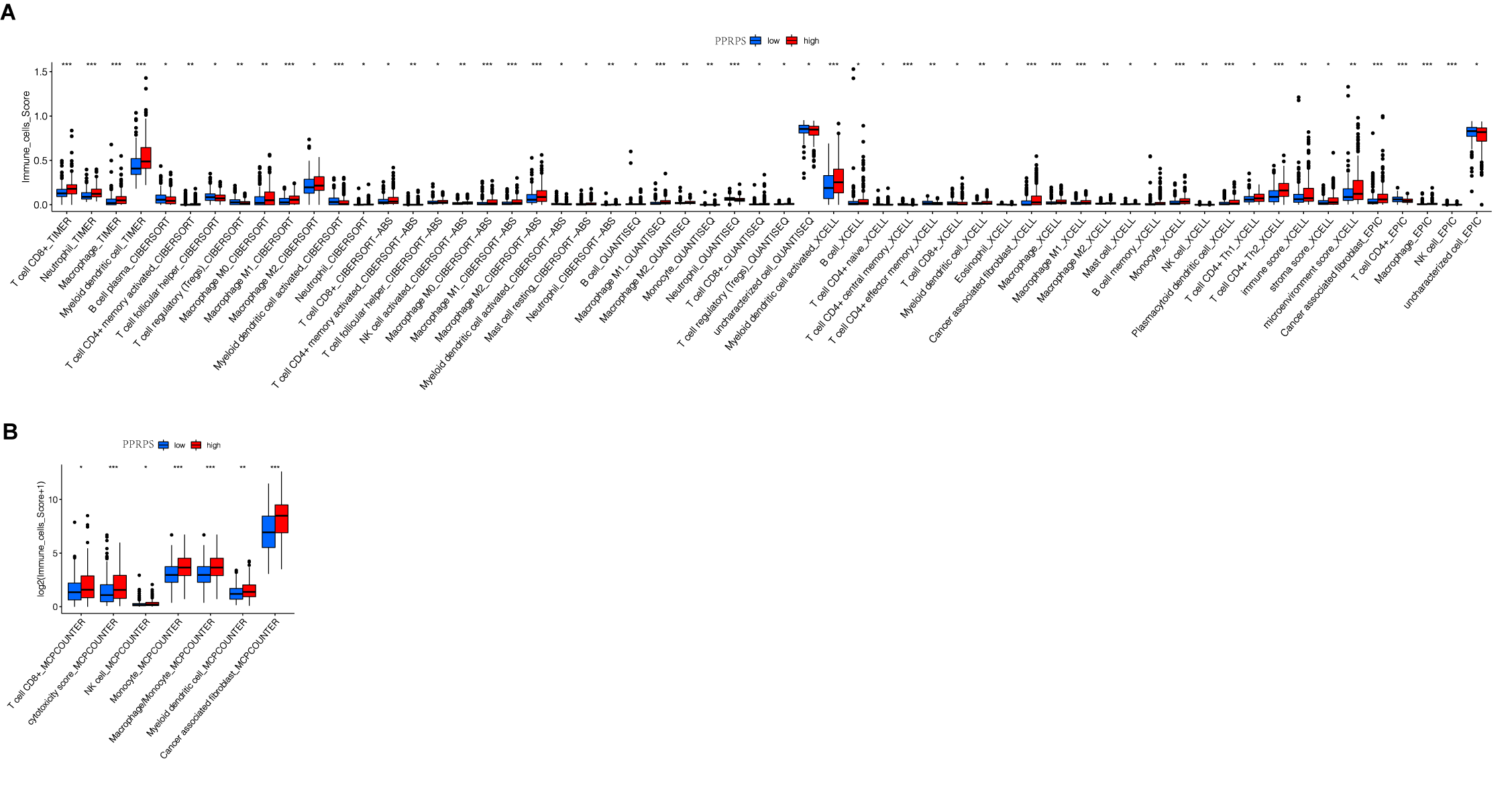

Supplement: Supplementary Figure 1 — Summary of immune cell scores of each type of immune cell between high- and low-PPRPS groups based on 7 algorithms. *p < 0.05, **p < 0.01, ***p < 0.001. [file Image_1.tif]

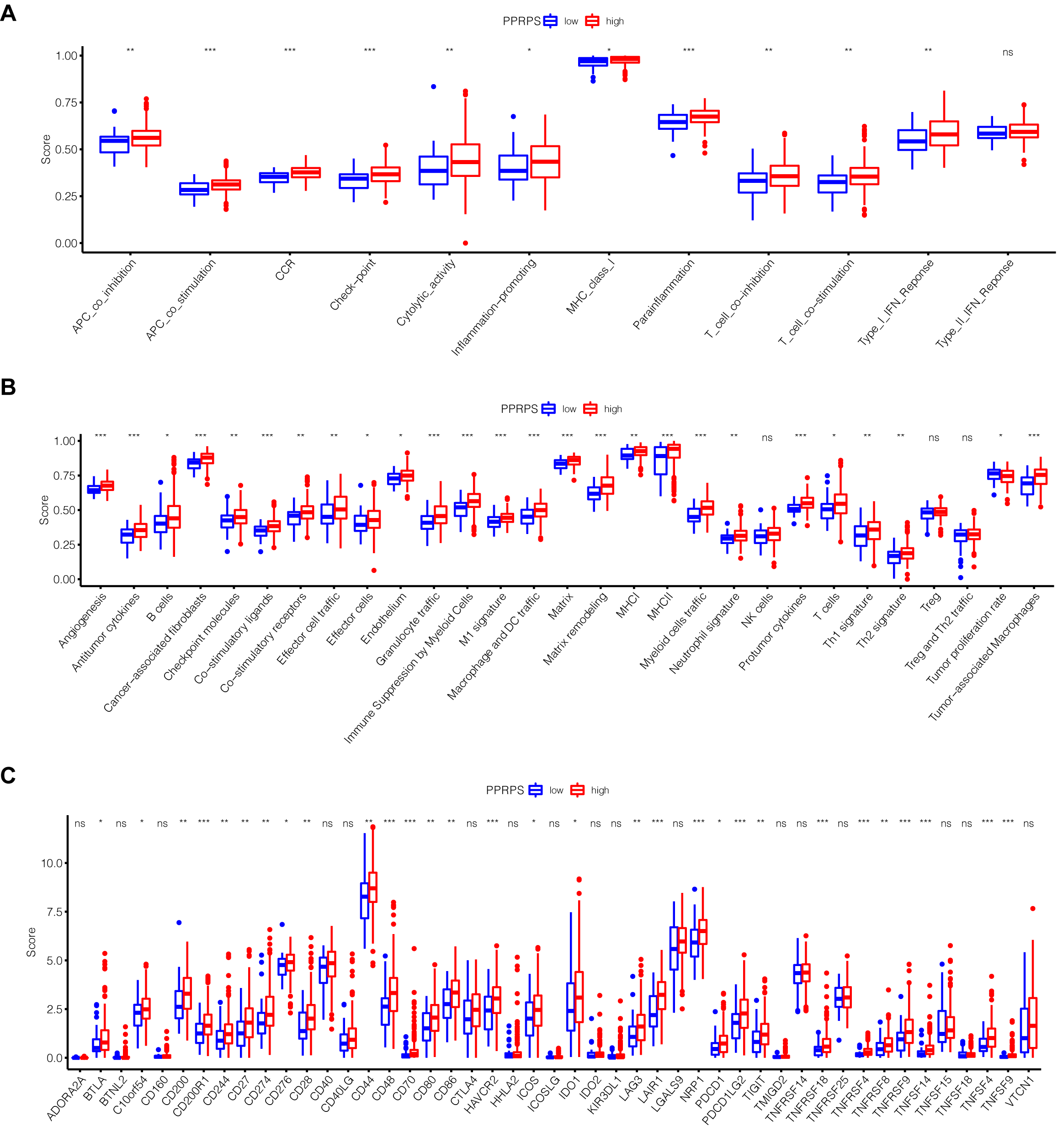

Supplement: Supplementary Figure 2 — Immune microenvironment, immune-related pathways, tumor microenvironment (TME) and checkpoint genes in the low- and high-PPRPS groups of the IMvigor210 cohort. (A) Scores of 13 immune-related pathways in the low- and high-PPRPS groups. (B) Twenty-nine functional gene expression signatures describing the TME in the low- and high-PPRPS groups. (C) Scores of immune checkpoint genes in the low- and high-PPRPS groups. *p < 0.05, **p < 0.01, ***p < 0.001. [file Image_2.tif]

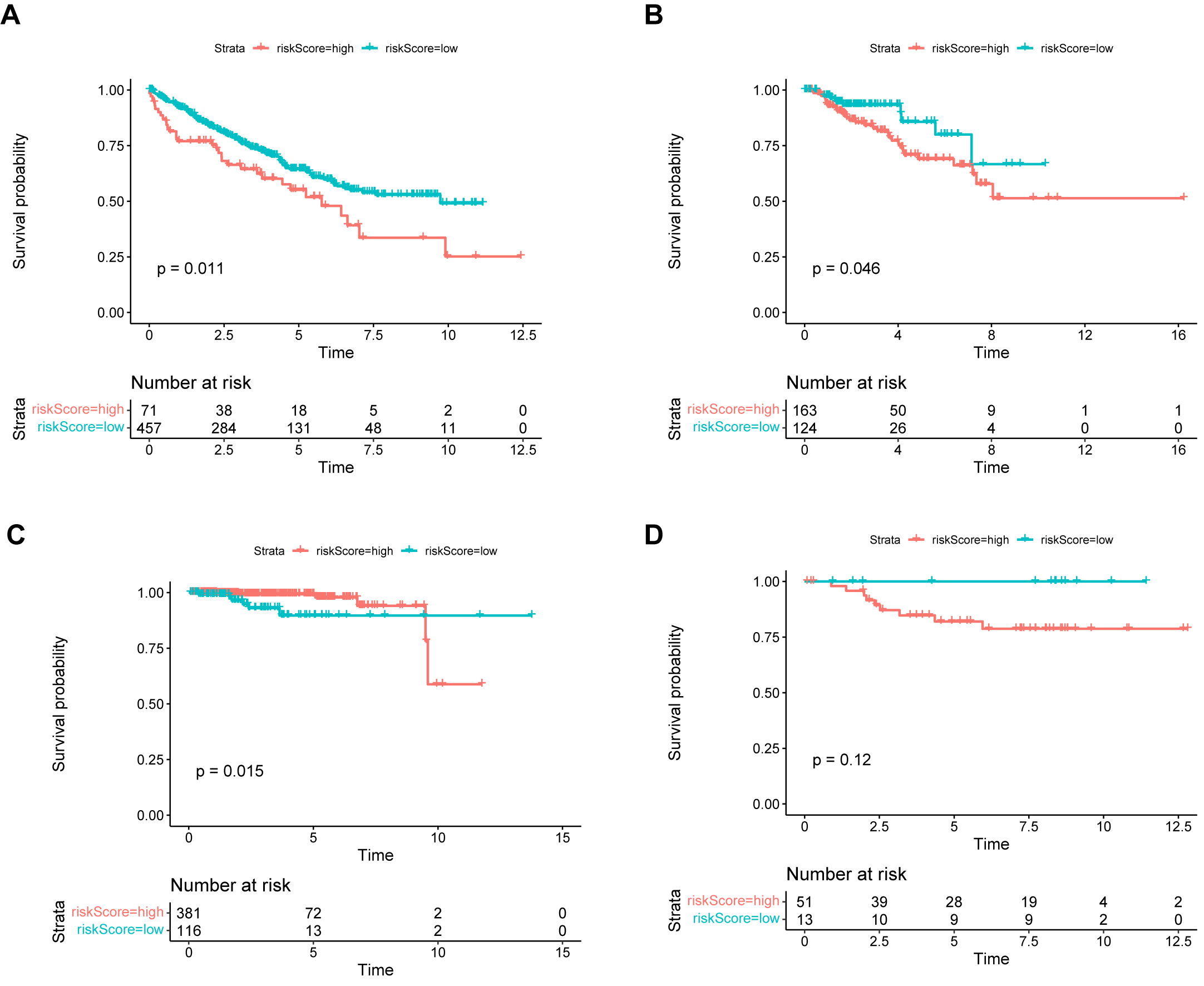

Supplement: Supplementary Figure 3 — Validation of the Pan-PCDs’ effect on the prognostic value of PPRPS in kidney cancer and prostate cancer. Kaplan–Meier (K-M) curve of the high- and low-PPRPS groups in kidney renal clear cell carcinoma (KIRC) (A), kidney renal papillary cell carcinoma (KIRP) (B), and prostate adenocarcinoma (PRAD) (C) demonstrating a great prognosis in patients. (D) the PPRPS did not show a good prognostic value to kidney chromophobe (KICH). [file Image_3.tif]
